# Detection of plane in remote sensing images using super-resolution

**DOI:** 10.1371/journal.pone.0265503

**Published:** 2022-04-21

**Authors:** YunYan Wang, Huaxuan Wu, Luo Shuai, Chen Peng, Zhiwei Yang

**Affiliations:** 1 School of Electrical and Electronic Engineering, Hubei University of Technology, Wuhan, China; 2 Xiangyang Industrial Institute of Hubei University of Technology, Wuhan, China; University of Bradford, UNITED KINGDOM

## Abstract

The object detection of remote sensing image often has low accuracy and high missed or false detection rate due to the large number of small objects, instance level noise and cloud occlusion. In this paper, a new object detection model based on SRGAN and YOLOV3 is proposed, which is called SR-YOLO. It solves the problems of SRGAN network sensitivity to hyper-parameters and modal collapse. Meanwhile, The FPN network in YOLOv3 is replaced by PANet, shortened the distance between the lowest and the highest layers, and the SR-YOLO model has strong robustness and high detection ability by using the enhanced path to enrich the characteristics of each layer. The experimental results on the UCAS-High Resolution Aerial Object Detection Dataset showed SR-YOLO has achieved excellent performance. Compared with YOLOv3, the average precision (AP) of SR-YOLO increased from 92.35% to 96.13%, the log-average miss rate (MR^-2^) decreased from 22% to 14%, and the Recall rate increased from 91.36% to 95.12%.

## 1 Introduction

Remote sensing image object detection is widely used in civil and military fields, such as guiding fruit picking, traffic management, environmental analysis, military surveying and mapping, and military object reconnaissance. Compared with the field survey, remote sensing image is more accurate. because it can capture ground information in real-time and obtain detailed information [1]. It can accurately recognize planes, ships, cars, and other objects in remote sensing images and has great significance in military operations and traffic management [2]. A method combining improved image resolution with object detection is proposed to improve the detection task of some low-resolution images. [3], regularization parameters were used by S2R2 to apply super-resolution technology to low-resolution face recognition. In [4], the translation invariance and global method were used in feature extraction. The artifacts and discontinuities in the low-resolution image are eliminated, and the face image is reconstructed with super-resolution to improve the detection accuracy. In addition, in some detection tasks, the accuracy of model detection is improved by deblurring the image [5–8] or denoising [9]. These methods improve the resolution on the basis of traditional image processing techniques, but due to their own limitations, they are still affected by a large number of small objects, instance level noise and cloud occlusion, so it is difficult to be applied to remote sensing image object detection.

This paper investigates a super-resolution approach that uses the power of end-to-end training in deep learning to combine low-level and high-level visual objects to produce what we call "You only see once with Super-resolution" (SR-YOLO). Super-resolution images contain more distinguishable features that can improve the accuracy of object detection. As a means of increasing the robustness of object detection to low-resolution inputs, this approach provides results substantially better than other object detection methods and is potentially applicable to a broad range of remote sensing satellite image processing tools and high-level tasks. Compared with previous studies, this paper uses advanced SRGAN super-resolution and the Third Version of You Look Only Once (YOLOv3) object detection, combines the two to apply to the plane detection in remote sensing satellite images, and improves their network structure to better apply for the detection of remote sensing images. SR-YOLO first solves the problem of SRGAN hyper-parameter sensitivity and pattern collapse and then enriches the semantic information of small objects through PANet [10]. Finally, super-resolution technology is used to drive the detector for object detection, which solves the problem of difficult remote sensing small object detection.

This paper is divided into two parts for improvement: 1) Improve the SRGAN network. The residual network is used to replace the normalized layer of the generated network and a penalty mechanism is added to reconstruct the loss function of the discriminator and generator. Our SRGAN network training process is more stable, obtains a more comprehensive feature space, and the generated images are more fine-grained. 2) Improve the YOLOV3 network. First, according to the dataset we use, we redefine a set of new bounding boxes suitable for plane detection. Finally, Path Aggregation Network (PANet) is used instead of Feature Pyramid Networks (FPN) [11] as the neck network to introduce subsampling, pool the features of all levels, shorten the distance between the bottom and the top, and use the enhancement path to enrich the features of each level.

This paper will introduce our proposed method from five chapters. The first chapter introduces the research background, existing problems and our solutions, and introduces the structure and outline of the paper. In Chapter II, the work related to super resolution and object detection is introduced. Chapter III describes our method in detail. Chapter IV presents the experimental process, including comparison with other algorithms and analysis of experimental results based on UCAS-AOD benchmark dataset. The Chapter V concludes the contributions and shortcomings of this paper.

## 2 Related work

At present, a considerable number of studies have been conducted to improve the detection accuracy of low-resolution images through image reconstruction. In contrast, binding by the super-resolution reconstruction and object detection task, relatively little research has been conducted to improve the detection accuracy of the remote sensing image. We review the work in two directions.

### 2.1 Image super resolution

Various super-resolution networks, including Super-Resolution Generative Confrontation Network (SRGAN), Enhanced Deep Super Resolution (EDSR), Deep Back Projection Network (DBPN), Super Resolution DenseNets, and Deep Laplacian Pyramid Network (DLPN) have been proposed [12–16]. These super-resolution networks show significant image magnification and greatly improve visual perception. These networks are more suitable for images with complex backgrounds. For example, [17] low-resolution images are used to perform super-resolution reconstruction through DBPN, and then send to the SSD detection network to improve the accuracy of complex background image detection. Super-resolution technology has also developed rapidly with the introduction of more efficient convolutional neural networks (CNN). Super-Resolution Convolutional Neural Network (SRCNN) [18] first uses bicubic interpolation to enlarge the low-resolution image to the object size, then fits the nonlinear mapping through a three-layer convolutional network, and finally output high-resolution image results. The network structure of SRCNN is very simple, with only three convolutional layers used. Some studies improved SRCNN, [19, 20] by introducing residual networks. [21] introduced recursive layers, but the use of hand-crafted layers for data enhancement still has limitations. DRRN [22] was inspired by [21, 23] and adopted a deeper network structure to obtain performance improvements. EDSR removes the redundant modules of Super-Resolution ResNet (SRResNet) [12] so that the size of the model can be increased to improve the quality of the results. Although the deep features of Diffusion-Convolutional Neural Networks (DCNN) can retain the realistic texture of high-frequency images, eliminating blurring and artifacts remains difficult, and this problem has been solved by [24] introducing perceived loss, while [25] introduced against losses. SRGAN uses perceptual loss and adversarial loss to improve the realism and fine texture details of the generated pictures. However, SRGAN has hyper-parameter sensitivity and mode collapse, leading to instability in the training process. At present, few super-resolution technologies are combined with remote sensing images to solve the problem of object detection in remote sensing images [26].

### 2.2 Object detection on remote sensing images

Object detection is divided into two-stage and one-stage categories. The two-stage detection algorithm divides the problem of object detection into two stages: generating Region Proposals and classifying and refining the candidate frame area [27–30]. One-stage detection algorithms are based on regression methods that do not need to generate the Region Proposals stage; a complicated framework is not necessary to directly obtain the category probability and position coordinate value of the object [31–34]. Generally, the detection accuracy of the two-stage algorithm is high and the speed is slow, which are suitable for scenes with high precision requirements. The detection accuracy of the one-stage algorithm is low and the speed is fast, which can realize real-time detection [35].

To improve the detection accuracy of objects in remote sensing images, [36] proposed a bounding box regression (USB-BBR) algorithm based on unsupervised scores, and combined the non-maximum suppression algorithm to optimize the bounding box of the detected object area. To address the small objects in large-scale and large scenes of remote sensing images, [37] proposed the Tiny-Net object detection method, which consists of the backbone Tiny-Net, the intermediate global attention block, the final classifier, and the detector. For detection of specific objects in remote sensing images, this model [38] trains multiple detectors, each of which is used particularly for buildings of a specific size. In addition, this model implicitly utilizes context information by simultaneously training road extraction tasks and building detection tasks. [39] proposed a new deep network-Rotatable Regional Residual Network (R3-Net), which is used to detect multi-object vehicles in aerial images and videos.

To improve the efficiency and accuracy of plane detection in remote sensing images, [40] proposed a weakly supervised learning framework to plane detection based on coupled CNNs. [41] presents an end-to-end semi-supervised object detection approach, in contrast to previous more complex multi-stage methods. The end-to-end training gradually improves pseudo label qualities during the curriculum and the more and more accurate pseudo labels in turn benefit object detection training. [42] presents a hybrid variable-wise weighted stacked autoencoder (HVW-SAE) is developed to learn quality-related features for soft sensor modeling. With the constraint of preferential reconstruction for more quality-related variables, it can ensure that the learned features contain more information for quality prediction. [43] propose a novel and flexible backbone framework, namely CBNetV2, to construct high-performance detectors using existing open-sourced pre-trained backbones under the pre-training fine-tuning paradigm. [44] present a novel dynamic head framework to unify object detection heads with attention. the proposed approach significantly improves the representation ability of object detection heads without any computational overhead. [45] propose spectral-spatial weighted kernel manifold embedded distribution alignment (SSWK-MEDA) for remote sensing image classification. The method applies a novel spatial information filter to effectively use similarity between nearby sample pixels and avoid the influence of non-sample pixels and utilize the geometric structure of features in manifold space to solve the problem of feature distortions of remote sensing data in transfer learning scenarios.

## 3 The proposed method

In this paper, a new detection model SR-YOLO is proposed. We explore a better combination of super-resolution SRGAN and YOLOv3 detection networks. So, firstly, we have to solve the problem of the unstable training process of the SRGAN network and improve the quality of the generated image. Second, the ability to detect small objects by YOLOv3 is important. Therefore, this section will be divided into two parts to introduce our improvements, namely SRGAN network improvement and YOLOv3 network improvement.

### 3.1 SRGAN network improvement

Generate network fine-tuning: First, the BN layer in the SRGAN generation network is replaced with a residual network. [13, 46] proves that, in PSNR-oriented tasks, removing the BN layer can improve performance and reduce computational complexity. Meanwhile, removing the BN layer may be generated to enhance the stability of the network training and can strengthen the generalization capability of the network. After replacing the BN layer of each layer with 3 × 3 convolution kernel convolution and PReLU activation layer, increasing the depth and complexity of the network, the features after each convolution is fully used, and the edge feature processing of the generated network is improved.

Reconstruction loss function: [47] analyzed the reasons for the instability of GAN training, which is that the JS divergence in the GAN network cannot smoothly brighten the distance between the distributions when the distribution p and q do not overlap so that effective gradient information cannot be generated at this position, thus leading to mode collapse. We learn from [47] idea, and reconstruct discriminant and generating network loss function, the training process is more stable, speed up the convergence rate of loss.

#### 3.1.1 Generate network fine-tuning

We use the method of network interpolation to maintain the perceptual quality and eliminate artifacts and noise in GAN, Specifically, we first train a PSNR-oriented network GPSNR and then obtain a GAN-based network GGAN by fine-tuning. We interpolate all the corresponding parameters of these two networks to derive an interpolated model GINTERP, The parameters are shown in Eq 1:

$$\theta_{G}^{INTERP}=(1-\alpha )\theta_{G}^{PSNR}+\alpha \theta_{G}^{GAN}$$ (1)

where, $\theta_{G}^{PSNR}$ and $\theta_{G}^{GAN}$ are the parameters of GINTERP, GPSNR, and GGAN, respectively, and α ∈ [0,1] is the interpolation parameter. Experiments show that when α is 0.2, PNSR reaches an ideal level.

**Fig 1:**
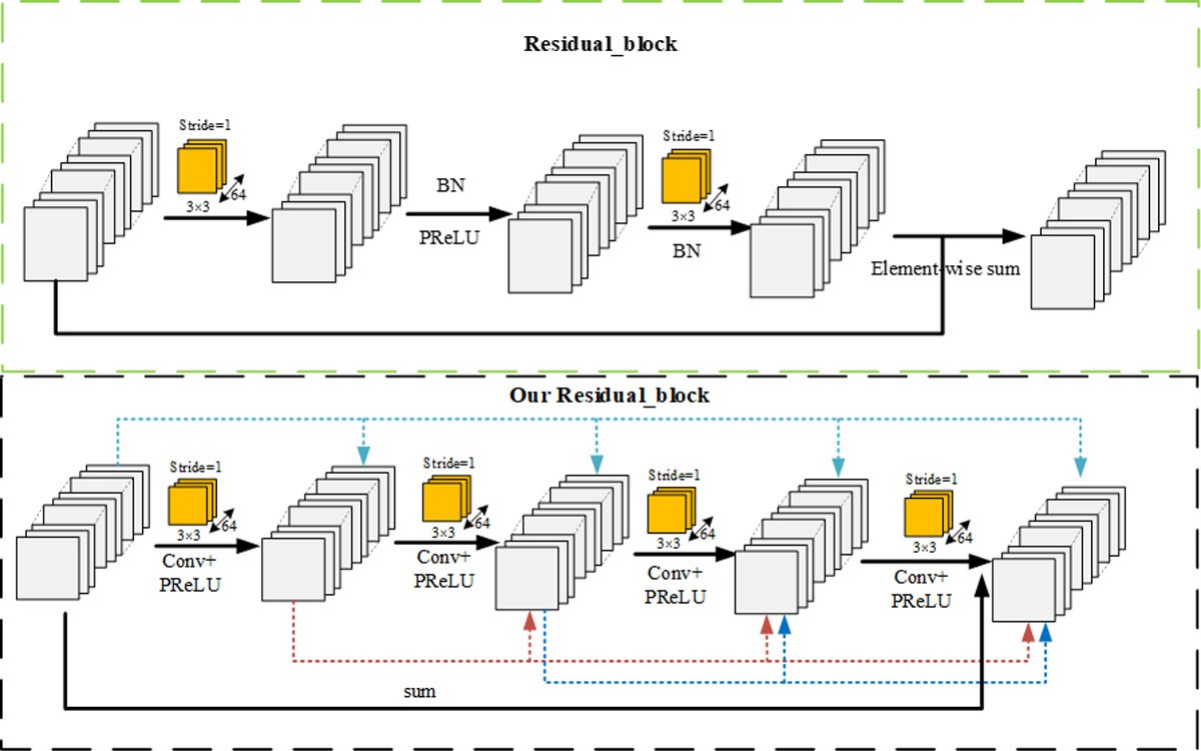
Residual block structure. Our Residual block replaces the BN layer of the original Residual block with a 3×3 convolution and PReLU activation layer; The upper part of the figure is the original residual block, and the lower part is our improved residual block.

We improve the residual block in the generating network. The residual block of the original generation network, as shown in the residual block in Fig 1, uses a 3×3 convolution kernel to convolve and BN layer, and then the PReLU function was selected to activate. Finally, the 3×3 convolution kernel convolution and normalization were carried out again. A very small number of parameters are added to the original residual block to make the feature information more abundant.

The feature vector obtained in the two convolution processes is combined with the original feature vector to ensure the completeness of the feature information. Sixteen original residual blocks are stacked in the generation network or a total of 16×2 BN layers. In super-resolution tasks, the output image and the original image are usually required to be consistent in color, contrast, and brightness, just change the resolution of the image and some details. However, the BN in the SRGAN generator stretches the contrast of the image, and the color distribution of the image after BN processing is also normalized, which destroys the original contrast information of the image and affects the quality of the output image. When the statistics of the training set differ from the test set, the BN layer will tend to generate bad artifacts and limit the generalization ability of the model. [44, 45] proved that in PSNR-oriented tasks, removing the BN layer can improve performance and reduce computational complexity. Meanwhile, removing the BN layer could enhance the stability of the network training and the generalization capability of the network. Therefore, as shown in our residual block in Fig 1, we replace the BN layer of the original residual block with a 3×3 convolution and PReLU activation layer, which increases the depth and complexity of the network, make full use of the features after each convolution, and the improvement of the edge feature processing of the generated network.

**Fig 2:**
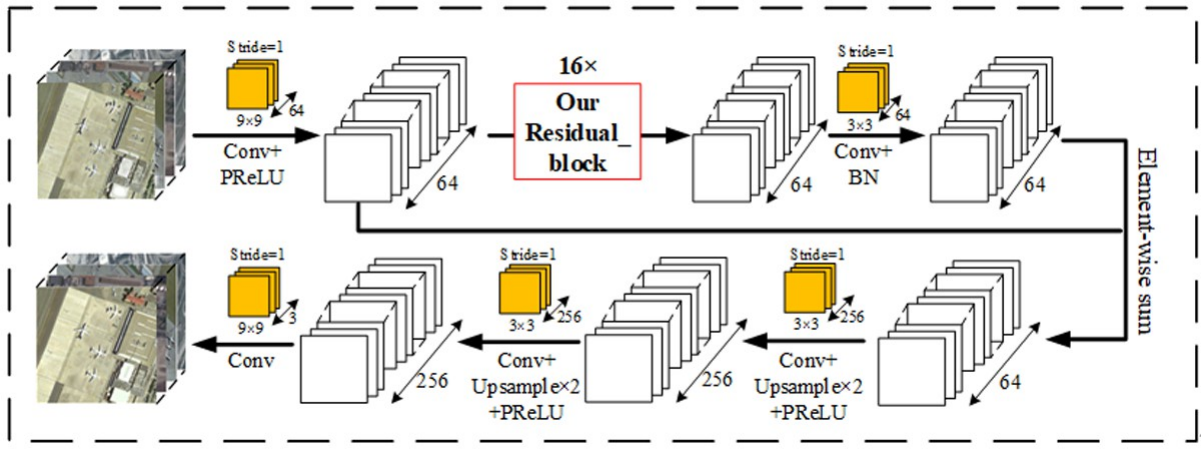
Generator network structure. We replace the original residual block with the new residual block (16×) and keep the rest unchanged.

In our generated network, as shown in Fig 2, 16 of our residual blocks were connected through a 9×9 convolution layer to obtain a complete underlying feature space. Then, two times the up sampling and PReLU activation were used. Finally, a 9×9 convolution layer was connected to restore the high-resolution remote sensing data.

#### 3.1.2 Reconstruction loss function

**Fig 3:**
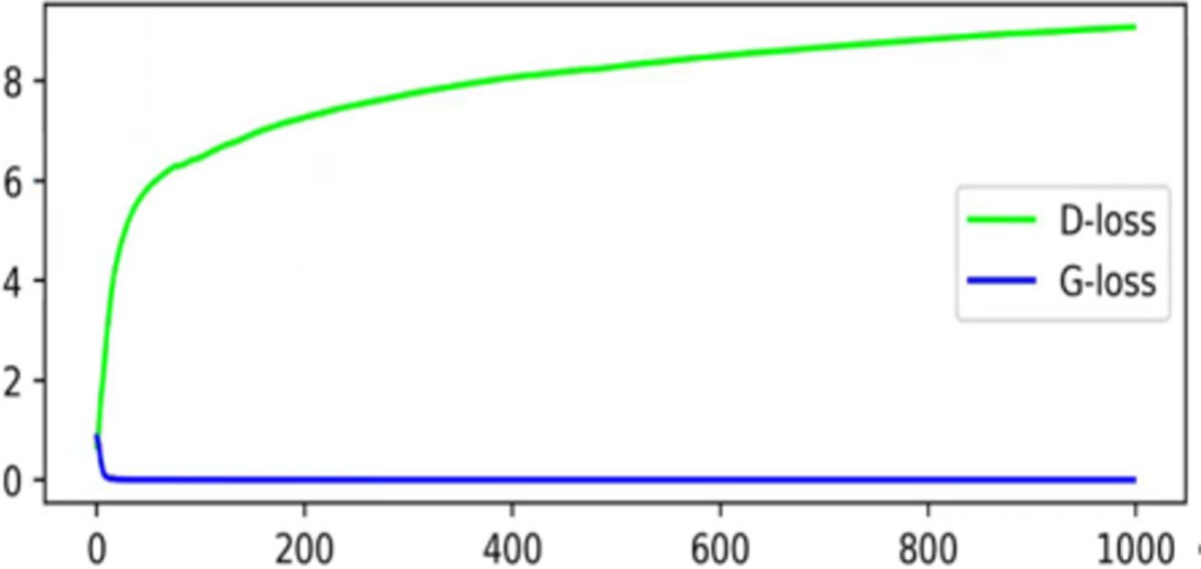
SRGAN training process appears mode collapse. D-loss is the generator loss, and G-loss is the generator loss. During the training process, the D-loss does not decrease in 1000 epochs.

By analyzing the reasons for the instability of GAN training [13], we find that when the JS divergence in the GAN network does not overlap the distributions p and q, the gradient surface is always 0, which leads to the inability to generate effective gradient information at this position and the gradients in training dispersion phenomenon. The parameters cannot be updated for a long time, leading to the collapse of the model and the failure of the network to converge. Fig 3 shows that the gradient dispersion appears in the SRGAN training process [47]. Introduced Earth-Mover Distance (EM distance) instead of JS divergence to solve the problem of instability in GAN training, and thus, we learned from this idea and introduced EM distance into the loss of the SRGAN discriminant network instead of cross-entropy.

The defect of JS divergence can be found in Eq 2. According to the definition of KL divergence and JS divergence, When θ is equal to zero, the distributions p and q overlap, and the JS divergence will change smoothly and produce effective gradient information.

However, when θ is not zero, no matter how long the distance between the distributions, the divergence of JS is a constant value log2, the reasoning process is shown in Eq (3–5). At this time, the JS divergence cannot generate effective gradient information. The gradient value is always 0, and the parameters of the generated network cannot be updated, resulting in difficulties in network training. By contrast, as the EM distance is shown in Eq 6, compared with the JS divergence, no matter how p and q are distributed, the EM distance always produces effective derivative information, which means that it is more suitable for guiding the training of the SRGAN network.


$$D_{JS}=(p\|q)=\{\begin{array}{c} \log2\;\theta \neq 0\\ 0\;\theta =0 \end{array}$$ (2)

$$D_{\mathrm{KL}}(p\|q)=\sum_{x=0,y∼U(0,1)}1\cdot \log\frac{1}{0}=+\infty$$ (3)

$$D_{\mathrm{KL}}(q\|p)=\sum_{x=\theta ,y∼U(0,1)}1\cdot \log\frac{1}{0}=+\infty$$ (4)

$$D_{\mathrm{JS}}(p\|q)=\frac{1}{2}(\sum_{x=0,y∼U(0,1)}1\cdot \log\frac{1}{1/2}+\sum_{x-0,y∼U(0,1)}1\cdot \log\frac{1}{1/2})=\log2$$ (5)

EM(p,q)=|θ| (6)

The effect of the discriminant is improved to make the discriminant network satisfy the first-order Lipschitz function constraint. Referring to the idea of [48], we introduce the penalty mechanism (GP) into the SRGAN discriminating network loss function. The definition of GP is as Eq 7, where $\overset{⌢}{x}$ is the sum of linear interpolation of the real picture *x*_*r*_ and the fake picture *x*_*f*_.


$$GP≜E_{\overset{⌢}{x}∼p\overset{⌢}{x}}[{\left(\|\nabla_{\overset{⌢}{x}}D(\overset{⌢}{x}){\|}_{2}-1\right)}^{2}]$$ (7)

At this time, the loss function of our SRGAN discriminant network is shown in Eq 8. It consists of the EM distance between the true and false images and the GP penalty term. Directly maximize the output value of the real sample, minimize the output value of the generated sample, and do not need to calculate the cross-entropy.


$$L(D)=E_{x∼P_{g}}[D(x_{f})]-E_{x∼P_{r}}[D(x_{r})]+\lambda E_{\overset{⌢}{x}∼P_{\overset{⌢}{x}}}[{\left(\|\nabla_{\overset{⌢}{x}}D(\overset{⌢}{x}){\|}_{2}-1\right)}^{2}]$$ (8)

We also redefine the loss function of the SRGAN generator as shown in Eq 9. It is composed of the maximum output value *L*_gen_ of the generated sample in the discrimination network, and the pixel-level mean square error *L*_*MSE*_ between the real picture *r* and the fake picture *x*_*r*_.


$$L(G)=L_{MSE}+10^{-6}L_{gen}$$ (9)

**Fig 4:**
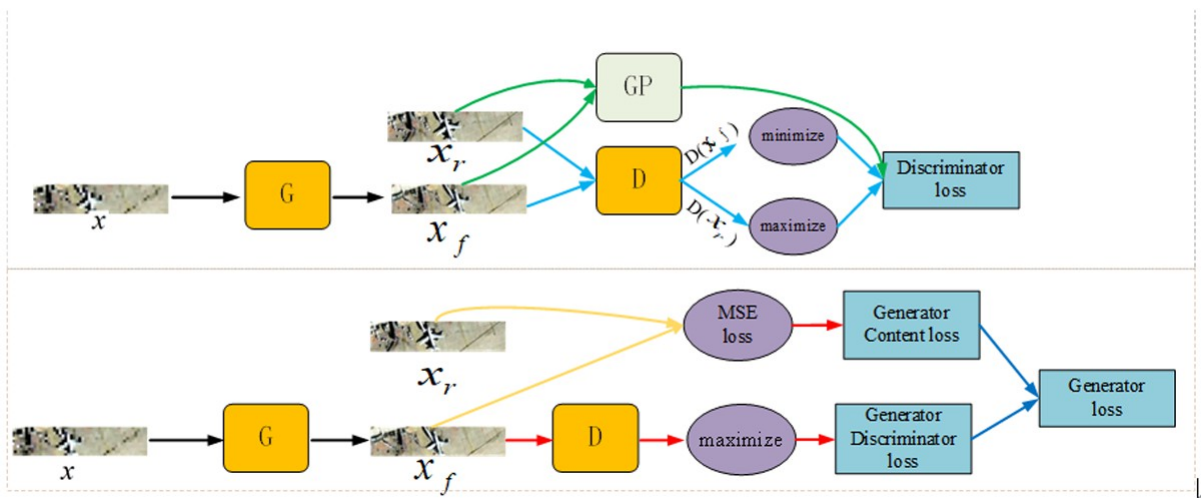
Visualization of our loss of function. The upper part of the figure is the loss function flow of the discrimination network; The next part is the loss function flow of the generated network.

In the training phase, we get *x*_*f*_ from *x*_*r*_ with down-sampling factor *r*, where *r* = 4. For one image, its channel number is C (C = 3), we described the size of *x* as *W*×*H*×*C*, so the size of corresponding *x*_*f*_ is *rW*×*rH*×*C*. Visualization of our loss of function is shown in Fig 4. In Fig 4, G is the generating network, D is the discriminant network, and GP is the penalty item.

**Fig 5:**
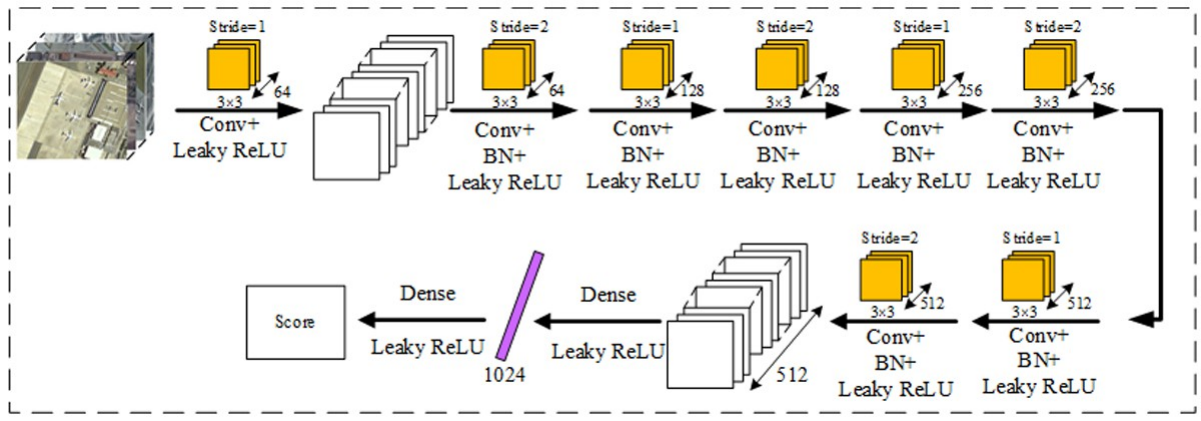
Our discriminant networks. We replace the last layer of the Sigmoid function with Leaky ReLU, and the rest is consistent with the original network.

In the original SRGAN discriminant network, the Sigmoid function is added to the last layer of the network to obtain the probability of each category. However, we use the discriminant network in the SRGAN network to measure the EM distance, and we replace the last layer of the Sigmoid function with the Leaky ReLU function. Our discriminative network structure is shown in Fig 5.

### 3.2 Yolov3 network improvement

**Fig 6:**
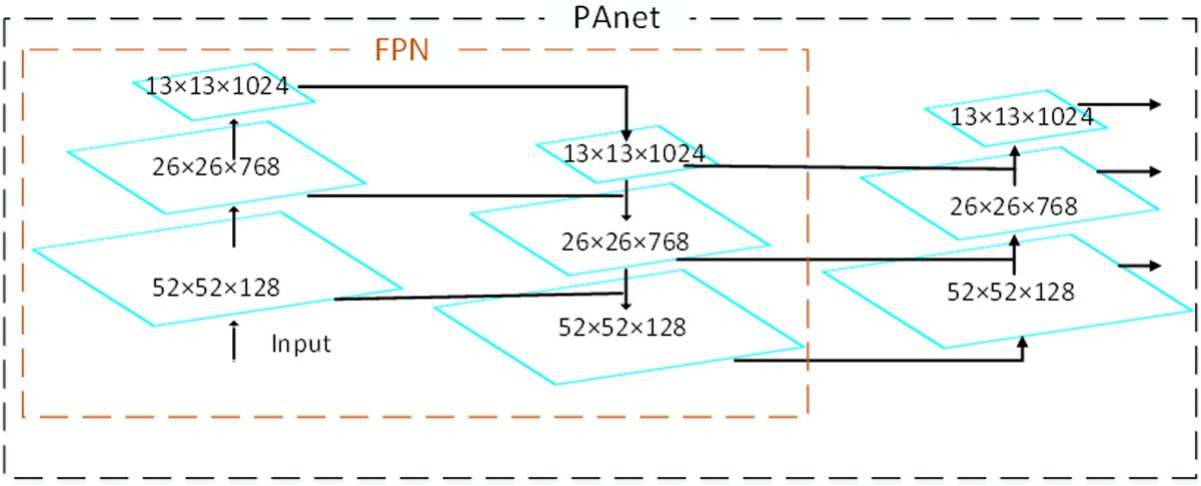
FPN and PANet network structure.

We improve the detection network YOLOv3 and replace FPN on the YOLOv3 neck with PANet. As shown in Fig 6, the path between the high-level features of FPN 52×52×128 and the low-level features 13×13×1024 is longer, which make it more difficult to accurate position information.

To shorten the information path and enhance the feature pyramid with low-level accurate positioning information, PANet creates a bottom-up path enhancement based on FPN. It is used to shorten the information path (shown by the green dotted line in Fig 6) and use the precise positioning signals stored in the low-level feature to improve the feature pyramid architecture. In addition, because our data set includes small and medium plane objects, so we are different from [49]. We do not introduce the SPP network into YOLOv3. to avoid the loss of location information and the introduction of noise caused by maximum pooling. Experiments show that our improvement enhances its ability to detect small objects. At the same time, to adapt to the planes of our dataset, we reclustered nine new bounding boxes through the K-means algorithm. The assignment of the nine new bounding boxes is shown in Table 1.

**Table 1:** Assignment of nine new bounding boxes by K-means.

52×52 layer	(11, 13)	(17, 30)	(31, 23)
26×26 layer	(30, 61)	(62, 45)	(59, 119)
13×13 layer	(178, 51)	(148, 95)	(18, 200)

## 4 Experiment

In this section, all kinds of the experimental settings will be introduced and the detection results of all related methods will be analyzed.

### 4.1 Datasets and platform of experiment

1) Introduction of datasets
  Very few remote sensing image data sets are suitable for object detection. After consideration, we choose the UCAS-AOD dataset for experimental comparison. UCAS-AOD Dataset: The UCAS-AOD Dataset contains 1,000 aerial images of the plane and 7,482 plane objects. The dataset contains challenging images, such as large clusters of small objects with instance noise and cloud occlusion. Our SR-YOLO will use these challenging remote sensing images to compare experiments with the most advanced detection algorithms.

2) Experimental environment
  Our experimental environment platform is as follows: GPU is NVIDIA RTX2060, CUDA is version 10.0, CUDNN is version 7.6.5, and PyTorch is version 1.2.0. The detailed information on the hardware and software environment is shown in Table 2.

**Table 2:** Hardware and software environment.

OS	Win 10 64-bit	CUDA	10.0
CPU	Intel CPU i7-9750H	cuDNN	7.6.5
GPU	NVIDIA RTX2060 (6G)	PyTorch	1.2.0
Python	3.7.0	YOLOv3	Ultralytics

### 4.2 Evaluation indicators

1) Super-resolution related evaluation indicators
  To improve the super-resolution part of SRGAN and have a better evaluation of the effectiveness of the experimental results, spectral angle mapper (SAM), peak signal-to-noise ratio (PSNR), and structural similarity (SSIM) are used as evaluation indicators.
  In Eq (10), SAM is the angle between the two spectra, that is, the spectrum angle, and x and y are the spectral curves of the reference spectrum and the test spectrum, respectively, the smaller the values are, the higher the similarity between the test spectrum and the reference spectrum, the probability, and accuracy of classification will be. In Eq (11–13).
$${\mathrm{SAM}=\cos}^{-1}\frac{\sum xy}{\sqrt{\sum {\left(x\right)}^{2}\sum {\left(y\right)}^{2}}}$$ (10)
$$MSE=\frac{1}{h\cdot w}\sum {}_{k=1}^{3}\sum {}_{i=1}^{h}\sum {}_{j=1}^{w}{\left(x(i,j,k)-y(i,j,k)\right)}^{2}$$ (11)
$$PSNR=10\times \mathrm{lg}(\frac{{\left(2^{n}-1\right)}^{2}}{MSE})$$ (12)
SSIM=l(x,y)⋅c(x,y)⋅s(x,y) (13)
  channel number k, image length w, and width h, image brightness *l*(*x*,*y*), contrast *c*(*x*,*y*) and similarity *s*(*x*,*y*) are denoted. The larger the PSNR and SSIM values are, the smaller the image distortion will be and the better the effect.
2) Object detection related evaluation indicators
  We use four indicators: precision, recall, average precision (AP), and log-average miss rate (MR^-2^) to better evaluate and compare new object detection models [50]. In particular, we are the first to introduce MR^-2^ as an evaluation indicator for plane detection. This indicator focuses on false positives (FP) and false negatives (FN) and is more suitable for crowded plane scenes in remote sensing images. When the actual and predicted labels are “True”, we call this case is true-positive (TP). When the actual and predicted labels are “False”, we call it true-negative (TN). Then, false-negative (FN) denotes the situation that the actual label is “True” and the predicted label is “False”. False-Positive (FP) is the opposite.

MR^-2^ takes the corresponding MR values of nine FPPI points at equal intervals from 10^−2^ to 10^0^ in the logarithmic space and calculates the average value. The lower the value is, the better the result will be, as shown in Eqs (14) and (15).


$$FPPI=\frac{FP}{N}$$ (14)

$$MR=1-R=\frac{FN}{TP+FN}$$ (15)

Precision refers to the proportion of correctly predicted True tags in all predicted True tags, which range between [0,1]. For plane detection, high accuracy represents the high confidence of a certain type of plane that has been detected. The precision calculation is shown in Eq (16).


$$precision=\frac{TP}{TP+FP}$$ (16)

The recall represents the proportion of correctly predicted “True” labels in the total number of actual “True” labels and ranges between [0,1]. For plane detection, high accuracy represents the high confidence of a certain type of plane that has been detected. The precision calculation is shown in Eq (17).


$$recall=\frac{TP}{TP+FN}$$ (17)

The Average Precision (AP) value can be taken by the area under the Precision-Recall (P-R) curve. AP is an important indicator to measure whether the prediction frame and position of the model are accurate. The AP calculation is shown in Eq (18).


$$AP_{i}=\int_{0}^{1}P_{i}(R_{i})dR_{i}$$ (18)

F1 score. Considering that precision and recall are often contradictory, the F1 Score is used to comprehensively measure the quality of one algorithm. The F1 Score calculation is shown in Eq (19).

$$F_{1}=2\frac{PR}{P+R}$$ (19)

### 4.3 Experimental results on SRGAN

On the UCAS-AOD dataset, SRResNet has the worst effect. The main reason is that the deep network is relatively simple, and the feature dimension is too large to be refined. The SRGAN network aims at the large difference of the feature matrix after convolution. The difference of the feature matrix data is reduced, and the sharpness of the experimental result image improved by adding the BN layer after each layer of convolution. The object texture feature obtains a better description but the image brightness in the image is difficult to reach the same brightness as the object. In this paper, a new dense residual network is introduced in SRGAN to ensure a balance of image quality without introducing planes to guarantee the remote sensing image brightness reconstructed. The experimental data obtained are shown in Table 3.

**Table 3:** Experimental results of different algorithms on UCAS-AOD dataset.

Algorithm	SRResNet	SRGAN	Our SRGAN
SAM	1.56	1.48	**1.43**
PSNR	21.69	24.65	**28.54**
SSIM	0.59	0.60	**0.74**

**Fig 7:**
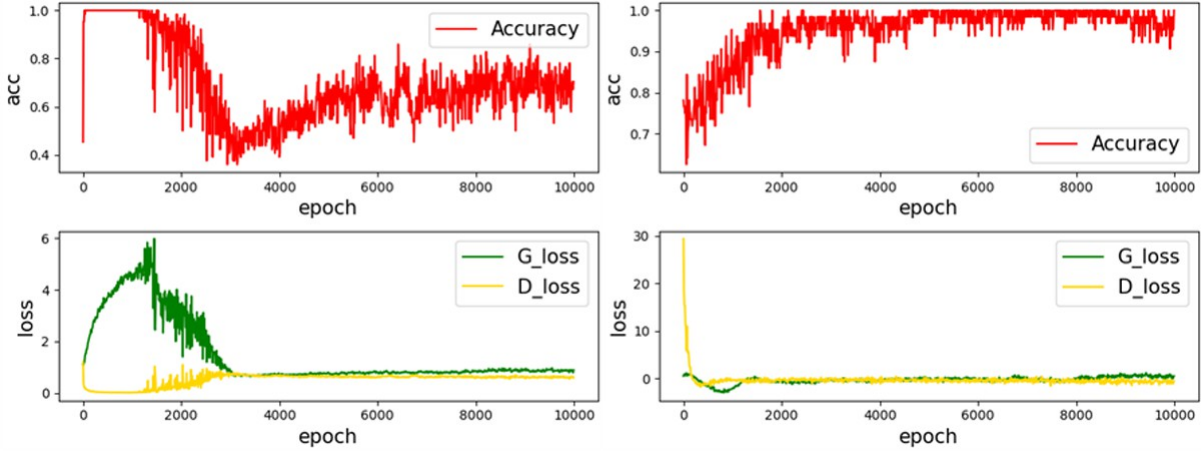
Original SRGAN and improved loss curve and accuracy curve. (a) SRGAN, (b) Our.

The image can be sent to the object detector for accurate recognition to realize each super-resolution image. Therefore, a more stable training process is required. The original SRGAN has the problem of mode collapse and hyper-parameter sensitivity. The resolution image quality is uneven. The training process becomes more stable after the original SRGAN loss function is improved. Fig 7 shows the loss curve and accuracy curve of the two networks.

**Fig 8:**
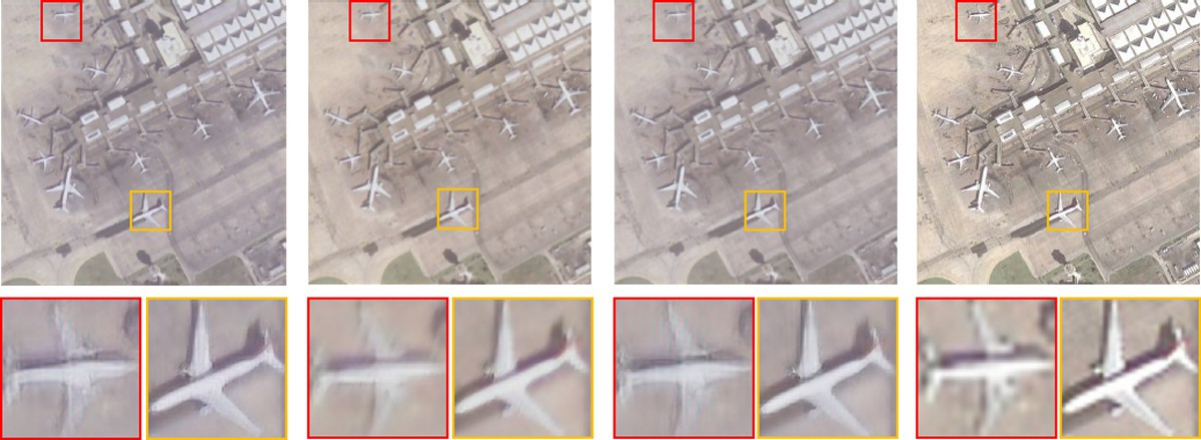
From left to right: SRResNet, SRGAN, our SRGAN, and the original HR image. The reconstructed image is four times the size of the original HR image.

The improved SRGAN network is used to perform super-resolution reconstruction of remote sensing images, increase the bit depth of the original image from 24 bits to 32 bits, enhance the semantic information contained in the object, improve the accuracy of the object detector, and reduce false detections rate and missed detection rate. The output image texture of the improved generation network is higher, and PSNR and SSIM are higher than the original SRGAN network. Fig 8 shows the comparison of super-resolution images.

### 4.4 Experimental results on SR-YOLO

In our SR-YOLO, we will generate a more stable generation network and higher-quality super-resolution plane images, and send them to the improved detection network. Experiments show the combination of the two networks is a good solution for remote sensing image objects. Greatly improve the detection effect.

We compare SR-YOLO with the current state-of-the-art object detection network. The comparison experiment is carried out on UCAS-AOD data, as shown in Table 4.

**Table 4:** AP of different detection algorithms on UCAS-AOD dataset (IOU 0.5).

Algorithms	Backbone	AP(%)
YOLOv3	DarkNet-53	92.35
SSD-512	VGG-16	89.50
RetinaNet	ResNet-101	95.18
Faster-RCNN	VGG-16	94.80
R-FCN	ResNet-101	94.99
R-FCN-OHEM	VGG-16	94.40
FPN	ResNet-101	91.03
RefineDet	VGG-16	94.20
YOLOv4	CSPDarkNet-53	96.02
YOLOv5-S	Focus	95.3
ConterNet	Hourglass	94.63
SR-YOLO	DarkNet-53	**96.13**

We have selected several state-of-the-art one-stage object detection algorithms and used multiple evaluation indicators for more detailed comparison experiments on the remote sensing data set UCAS-AOD. The experimental results are shown in Table 5.

**Table 5:** Comparative experiment of multiple networks on UCAS-AOD dataset (IOU 0.5).

Algorithms	F1(%)	R(%)	P(%)	MR^-2^(%)	AP(%)
YOLOv3	92	91.36	93.45	22	92.35
YOLOv4	94	**98.05**	90.96	18	96.02
YOLOv5-S	**95**	95	**96.73**	21	95.3
ConterNet	91	96.91	94.83	30	94.63
SR-YOLO	92	95.21	89.72	**14**	**96.13**

**Fig 9:**
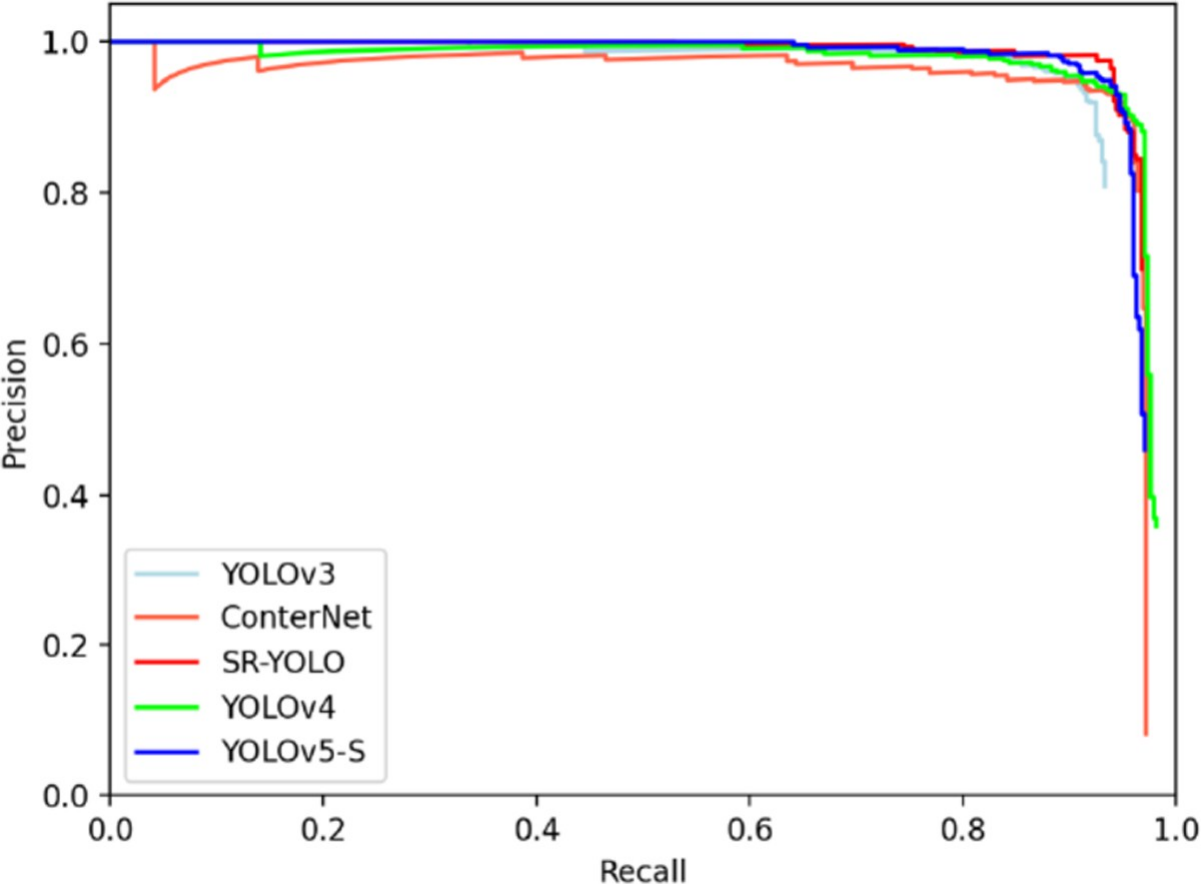
The AP curve of the algorithm in this paper and the current most advanced algorithm.

The above test results are plotted as a precision-recall rate curve (PR curve). The graph is shown in Fig 9. The overall detection performance of the SR-YOLO network can be seen from the curve. The detection performance is better than other one-stage networks.

The indicator of the network model in the ablation study is shown in Table 6. We proposed three improved modules in total. To verify the existence value of the proposed modules, we designed ablation experiments on our improved module by gradually adding improvements. Then, we trained and tested the models to obtain the MR^-2^ and mAP, and Recall. The order of adding the modules is as follows: K-means, PANet, and SR. During the training process, the dynamic process of training can be visually observed by drawing the loss curve.

**Table 6:** Specific index values of three networks.

Algorithms	R (%)	MR^-2^ (%)	AP (%)
YOLOv3(Step1)	91.36	22	92.35
Step1+k-means (Step2)	92.58	18	92.49
Step2+PANet (Step3)	95	18	93.79
Our (Step3+SR)	**95.21**	**14**	**96.13**

### 4.5 Application evaluation

In the USAS-AOD dataset, we selected three groups of plane images with challenging attributes, namely, large clusters of small objects, instance-level noise, and cloud occlusion. These images are an extremely challenging task for the detector.

**Fig 10:**
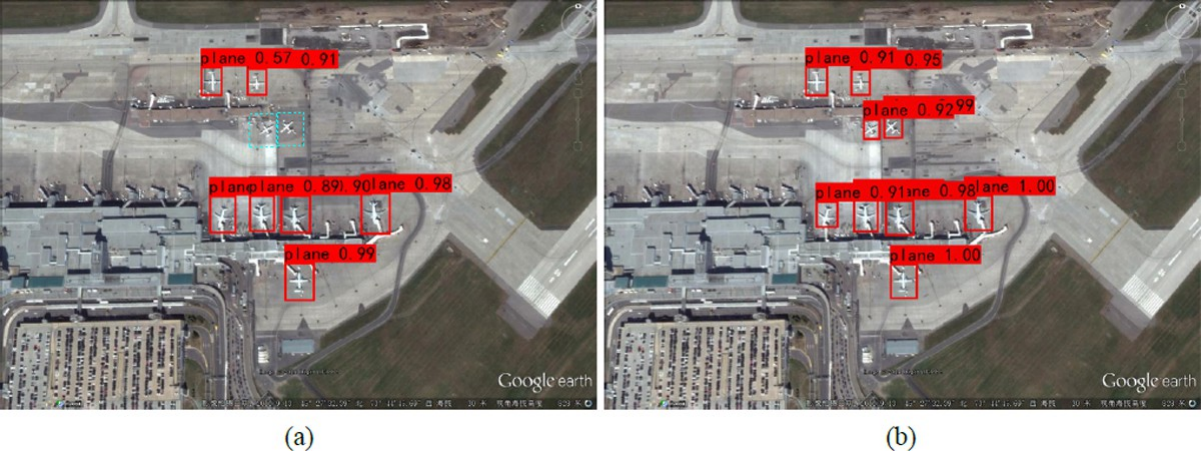
Test results of YOLOv3 and SR-YOLO. The cyan box indicates the missed plane object. (a) Results of YOLOv3 algorithm detection; (b) Results of SR-YOLO algorithm detection.

In a remote sensing image, the field of vision is relatively large, and the plane object is smaller than the image, making it difficult to detect it. To address this problem, our SR-YOLO detection network generates images and object images through super-resolution reconstruction, which could make them more similar in semantics and style. The original image was magnified by four times to enrich the semantic information contained in the small object of the plane, which allows the effective feature information to be retained after multiple subsampling. Then, the information was used for multi-scale feature fusion through PANet to enrich the features of each level. Fig 10 shows the detection results of SR-YOLO and YOLOv3 on six 416 pixels × 416 pixels airport images, with each image containing different small-pixel airplanes. Fig 10(A) shows the detection results of YOLOv3, while Fig 10(B) shows the detection results of SR-YOLO.

A comparison of the detection results of the same image shows that the plane with obvious features is accurately detected by the two networks. However, when the plane pixels in the image are small and the image features are not obvious, the YOLOv3 missed detection phenomenon is serious, while SR-YOLO avoids these mistakes perfectly. Therefore, compared with YOLOv3, SR-YOLO has stronger robustness, detection ability to recognize small objects, and higher accuracy.

**Fig 11:**
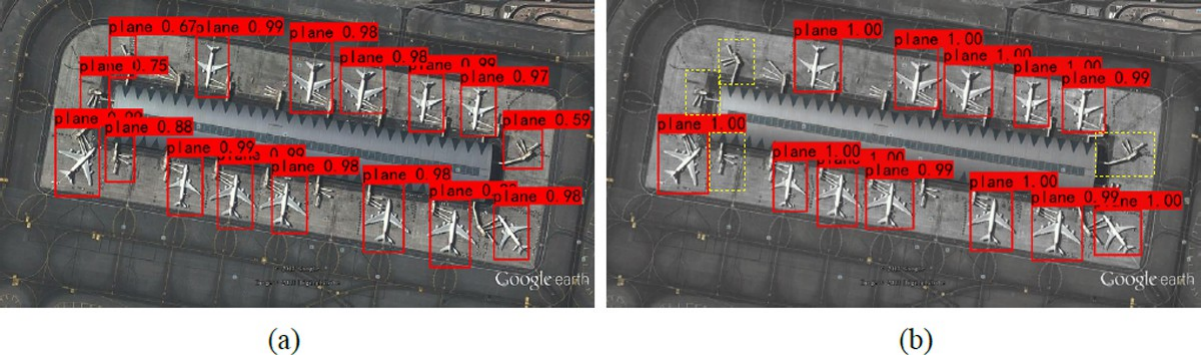
SR-YOLO and YOLOv3’s detection of plane images with instance-level noise, the yellow box marks the objects that the YOLOv3 network false detection as the plane. (a) The results of YOLOv3 algorithm detection; (b) The results of SR-YOLO algorithm detection.

Dense and small plane objects in remote sensing images often have instance-level noise, that is, the background interfering with object detection. The presence of instance-level noise is also the reason for the difficulty encountered in remote sensing image detection. For example, in Fig 11, the plane objects with similar shapes have a higher response in the feature map, inter-class and class-like feature coupling can be observed among dense plane objects. The problem with blurring the boundaries of internal features is that the response of the plane surrounded by the background is not prominent enough. The presence of instance-level noise can easily lead to false detection of the detector. Fig 11 shows the detection results of the plane in remote sensing images by the two networks. A comparison of the falsely detected plane objects as indicated in the yellow box in Fig 11 shows the object that YOLOv3 falsely detected as a plane. The false detection phenomenon of SR-YOLO is lighter than that of YOLOv3. For objects that YOLOv3 falsely detects as a plane, SR-YOLO did not recognize them as a plane, which fully proves that SR-YOLO is more capable of detecting planes in remote sensing images than the original YOLOv3.

**Fig 12:**
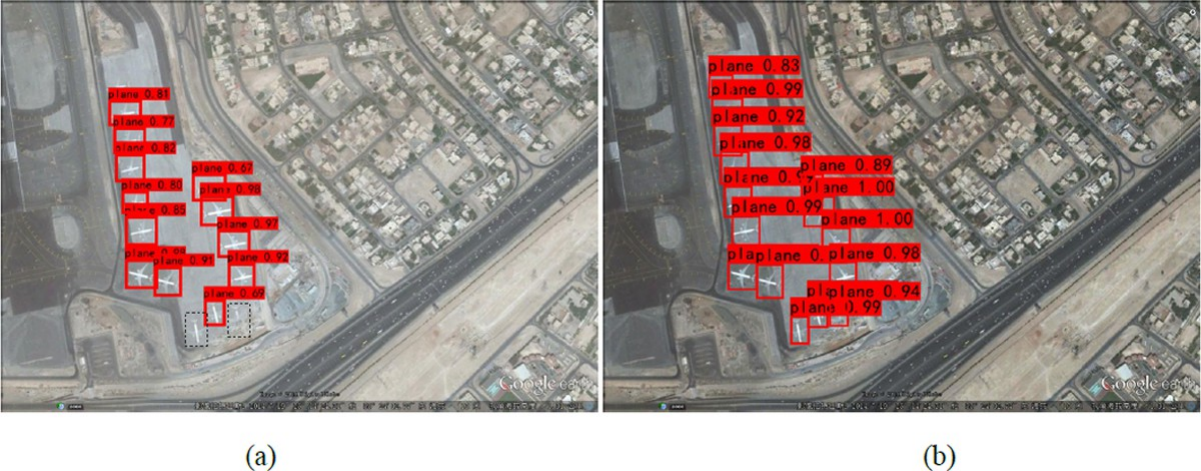
SR-YOLO and YOLOv3 detect plane images with cloud and fog occlusion. The black box indicates the undetected plane. (a) Results of YOLOv3 algorithm detection; (b) Results of SR-YOLO algorithm detection.

In the case of a complicated meteorological environment, the acquisition of remote sensing images is often not ideal, and cloud cover is one of the most frequently encountered situations. For example, in Fig 12, the plane covered by clouds will lose certain characteristic information that affects the plane recognition rate. Fig 12 shows that although SR-YOLO faces some omissions in the remote sensing image occluded by clouds and fog, the phenomenon is lighter than that of YOLOv3 and can detect most planes, which fully proves that SR-YOLO is more capable of plane detection in complex environments in remote sensing images than the YOLOv3.

This study makes statistics based on Figs 10–12, and obtains Table 7 to evaluate quantitatively the detection effect of these two networks on plane images with challenging attributes. For remote sensing images with large clusters and small objects, instance-level noise, and cloud occlusion, the recall, and precision of YOLOv3 are 73.77% and 93.75%, respectively, and the recall and precision of SR-YOLO are 96.61% and 100%, respectively. These indicators further prove that the SR-YOLO model has stronger robustness to remote sensing images, and indicate that the improvement of the model is effective.

**Table 7:** Comparison of detection performance of YOLOv3 and SR-YOLO on remote sensing images.

algorithms	TP	FP	TN	FN	Recall(%)	Precision (%)
YOLOv3	31	4	-	5	86	88
SR-YOLO	36	0	-	0	100	100

Although adding the auxiliary network will increase the computation of the whole network to a certain extent, the test results show that the detection speed of the whole network conforms to the actual application scenarios. The test environment was tested under NVIDIA 2060 graphics cards. The comparison of the test results is shown in Table 8. The evaluation index is FPS (Frames Per Second):

**Table 8:** Frames per second test comparison.

algorithms	FPS
YOLOv3	63
SR-YOLO	61

**Fig 13:**
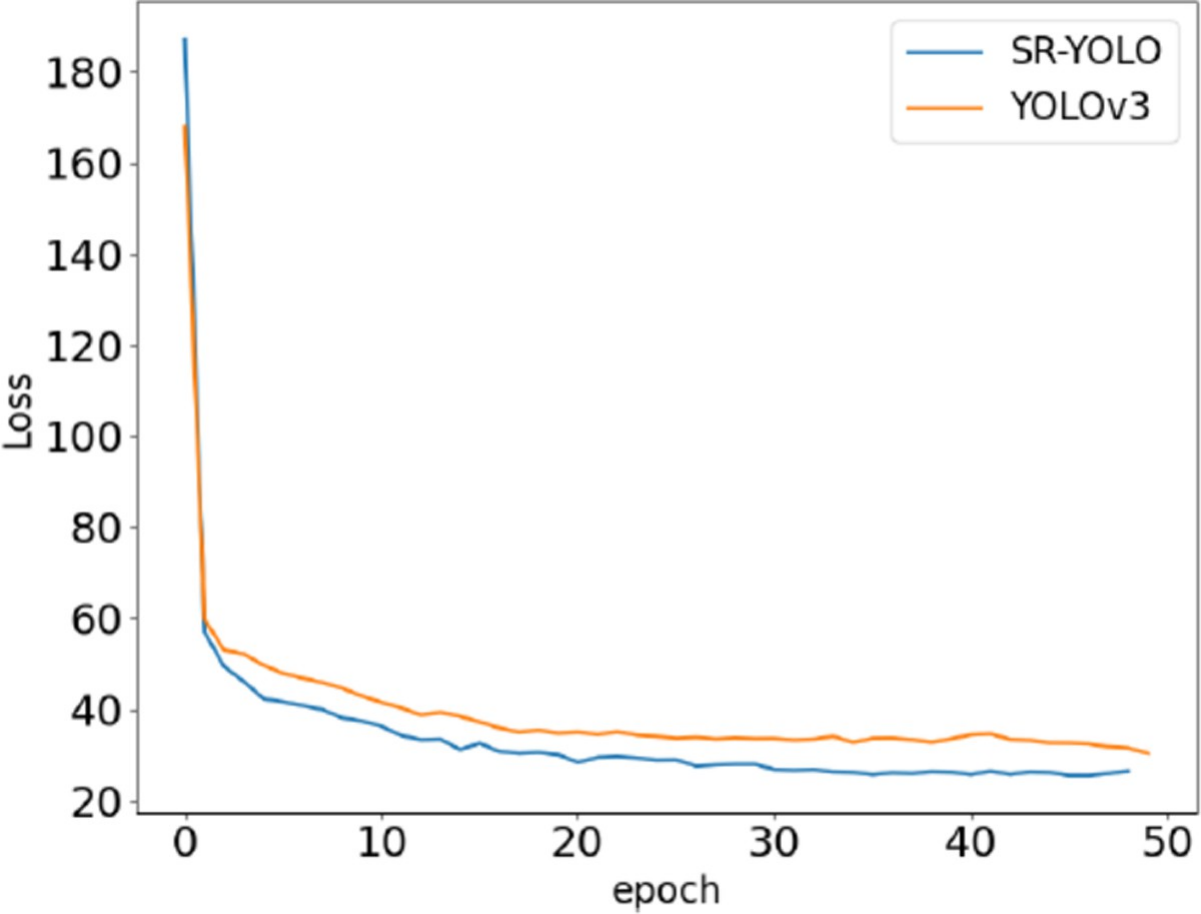
Training loss function graph.

Fig 13 shows the corresponding average loss curve during the training of the method model in this paper. The abscissa indicates the number of training iterations and the ordinate indicates the Loss value during training.

The SR-YOLO network was tested and compared with typical networks in one-stage and two-stage. The remote sensing dataset used in the previous experiment was still used to compare the average accuracy and processing time. The comparison results are shown in Table 9.

**Table 9:** Network test comparison.

algorithms	AP	FPS
Faster-RCNN	94.80	10
YOLOv5-s	95.3	65
SR-YOLO	**96.13**	61

Both Faster R-CNN and YOLOv5-s are networks that have performed well in recent years. YOLOv5-s belongs to a one-stage type network. From the above test results, SR-YOLO has Similar accuracy to YOLOv5-s and frames. While Faster R-CNN is a two-stage type network, SR-YOLO is only slightly higher than the AP by 1.33%, but the detection speed is faster. That is to say, SR-YOLO has a good performance in detection accuracy and detection speed.

To address the difficulty of detecting airplanes in remote sensing images, the improvement in this paper effectively improves the defects of YOLOv3 to detect airplanes in remote sensing images. For example, it improves the detection of airplanes in dense and small scenes with instance-level noise and cloud and fog occlusion. The detection accuracy of remote sensing plane objects reduces the missed detection rate and the false detection rate. At the same time, the test results show that the detection speed of the whole network conforms to the actual application scenarios.

## 5 Conclusion

In this paper, a new object detection model based on super-resolution network SRGAN and detection network YOLOV3 is proposed to solve the problem that the object detection of remote sensing image often has low accuracy and high missed or false detection rate. The specific contributions of this paper are summarized as follows:

1. the improvement of SRGAN. we replace the BN layer in the SRGAN generation network with a residual network. The BN layer is removed to improve the performance of the generation network and reduce the complexity of the calculation. At the same time, removing the BN layer can improve the stability of the generation network training and improve the generalization ability of the network. We also improved the loss function of the discriminant and the generation networks, solved the problem of mode collapse and hyper-parameter sensitivity in the SRGAN network training process, ensured the convergence speed of the loss, and made the training process more stable.
2. the improvement of YOLOv3, we replaced the FPN on the neck of YOLOv3 with PANet, shortened the distance between the lowest and the highest layers, and used enhanced paths to enrich the features of each scale. At the same time, to adapt to the plane detection dimension of our data set, we re-clustered nine new bounding boxes through K-means.

The improvement of the algorithm relies on the combination of SRGAN and YOLOv3, and the enhancement path is adopted to enrich the scale features, which leads to the increase of the memory occupied by the model, making the training consume more computing resources and reduce the real-time performance.

## Supporting information

S1 Dataset (TXT)
